# The response of the Oxford classification to steroid in IgA nephropathy: a systematic review and meta-analysis

**DOI:** 10.18632/oncotarget.19574

**Published:** 2017-07-26

**Authors:** Pingping Yang, Xi Chen, Lei Zeng, Hua Hao, Gaosi Xu

**Affiliations:** ^1^ Department of Nephrology, The Second Affiliated Hospital of Nanchang University, Nanchang, China; ^2^ Grade 2013, The Second Clinical Medical College of Nanchang University, Nanchang, China; ^3^ Department of Pathology, The Second Affiliated Hospital of Nanchang University, Nanchang, China

**Keywords:** IgA nephropathy, Oxford classification, steroid, proteinuria

## Abstract

**Background:**

The present review is aimed to evaluate the correlation between pathological features and the response to steroid in the patients with IgA nephropathy according to the Oxford classification, mesangial hypercellularity (M), endocapillary hypercellularity (E), segmental glomerulosclerosis (S), tubular atrophy and interstitial fibrosis (T).

**Methods:**

We searched Chinese Biomedical Database, EMBASE, Cochrane Library, PubMed and MEDLINE with all spellings of “IgA nephropathy”, “Oxford Classification”, and “steroid”.

**Results:**

5 studies with 637 patients were eligible for inclusion. The analysis showed that M1, S1, and T1/2 was strongly associated with the prediction to steroid resistance when compared with M0 [odds ratio (OR) 1.89, 95% confidence interval (*CI*) 1.01 - 3.56, *P* < 0.05], S0 (OR 2.24, 95% *CI* 0.99 - 5.08, *P* = 0.05) and T0 (OR 2.16, 95% *CI* 1.29 - 3.63, *P* = 0.004) respectively. There is no difference in steroid sensitivity between E0 and E1 (*P* = 0.55). The pooled OR of steroid resistance for E1 versus T1/2 is 0.50 (*P* = 0.04).

**Conclusion:**

IgA nephropathy patients with serious pathological changes (M1, S1, and T1/2) were more resistant to steroid than slight ones (M0, S0, and T0), and E1 is better response to steroid therapy than T1/2.

## INTRODUCTION

Immunoglobulin A nephropathy (IgAN) characterized by IgA deposition in the glomerular mesangium is the most common form of primary glomerulonephritis worldwide [1]. 15 to 20 percent of patients with IgAN develop end-stage renal failure (ESRD) within 10 years, and 30 to 40 percent within 20 years [2, 3]. IgAN is a type of immune complex-mediated glomerulonephritis with widely variable clinical courses and pathologic features. Histopathologic classification is the key to evaluate the severity of the lesions and to guide therapeutic strategies for IgAN in clinical practice [2, 4–7].

In 2009, the Oxford Classification of IgAN identified four histopathologic features of prognostic value: mesangial hypercellularity (M), endocapillary hypercellularity (E), segmental glomerulosclerosis (S), tubular atrophy and interstitial fibrosis (T) and abbreviated with MEST [8]. It has been validated in different populations, while the results remain inconsistent. A meta-analysis suggested that M, S, and T lesions, but not E score are associated strongly with the progression to kidney failure [9]. However, up to date, there is not any synthetic study to investigate the response of different MEST lesions to the steroids treatment.

The Kidney Disease Improving Global Outcomes (KDIGO) guidelines suggest that patients with persistent proteinuria ≥ 1.0 g/24h despite 3-6 months of intensive supportive care, and an estimated glomerular filtration rate (eGFR) more than 50 ml/min per 1.73 m^2^ accept a 6-month course of corticosteroids [10]. Whether the benefits of corticosteroids vary, depending on proteinuria, eGFR, or the differences in pathologic findings is uncertain, because that these issues have been rarely addressed by randomized controlled trials (RCTs). The predictive value of Oxford Classification of IgAN in some clinicopathological studies has also been confounded by steroid/immunosuppresion associated bias [11]. In the present study, for the first time, we performed a systematic review and pool the available validation data to evaluate the response of pathological changes to steroid therapy in patients with IgAN.

## RESULTS

### Trial flow and study characteristics

**Figure 1:**
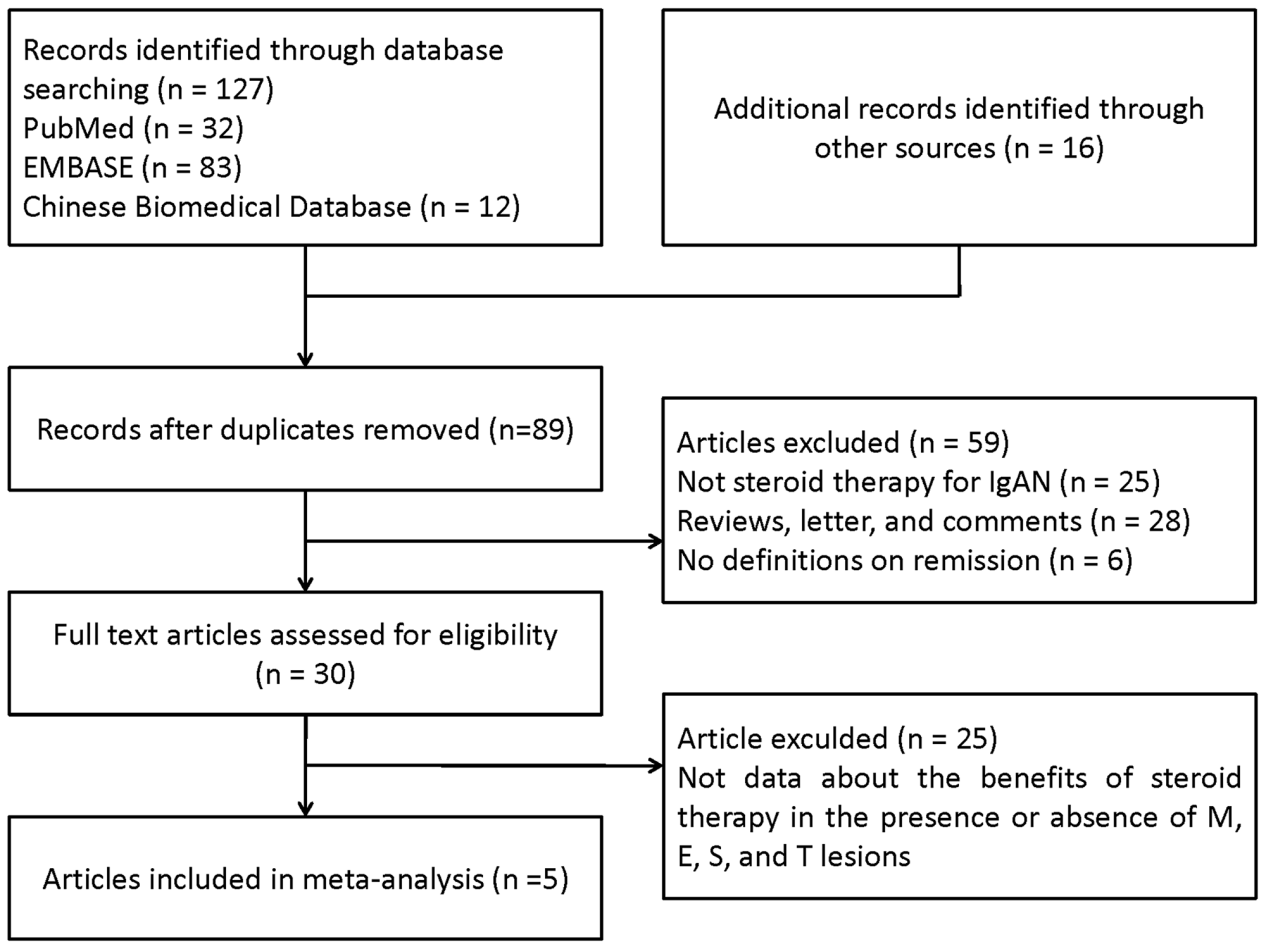
PRISMA flow diagram of identification process for eligible articles Results of literature search on Oxford Classification respond to steroid/immunosuppresion therapy in IgA nephropathy.

Our search strategy identified 89 articles, of which 5 met the criteria for full-text review. Five studies [13–17] including 637 patients were eligible for inclusion. Reasons for exclusion are listed in Figure 1. The characteristics of the included studies are summarized in the Table 1. The inclusion criteria used in two trials [13, 14] was IgAN patients with nephrotic syndrome, and the other one [15] was biopsy-proved IgAN and urinary protein excretion ranging from 1.0 to 3.5 g/24h. Another two studies [16, 17] did not limit the scope of proteinuria, and the last study [16] used the immunosuppressive therapy and was only provided the data of M1, E1, S1, T0, and T1/2, without M0, E0, and S0. As for M0, E0, S0 lesions, there were 4 studies including 240 patients were reported. And, as for the T lesions, 5 studies with 361 participants were reported the association with steroid/immunosupression therapy to IgAN. When compared with each other, for example, M1/E1, 5 studies were listed in the forest plot.

**Table 1:** Characteristics of studies included in the meta-analysis

Study	Subjects group	N	Group	Follow up (m)	Definition of remission	Quality score
**Shi SF 2011** [16]	IgAN	294	IS+RASB, RASB	12	Remission: proteinuria < 1.0 g/d	7
**Morigama T 2012** [13]	Nephrotic IgAN	42	Steroid, Non-steroid	48	Incomplete remission: proteinuria < 1.0 g/d	8
**Kang ZJ 2015** [14]	IgAN children with NS	58	Steroid, MMF	17	Remission: proteinuria < 0.3 g/gIncomplete remission: < 50% reduction in proteinuria	7
**Tesar V 2015** [17]	Children and adults with IgAN	184	Steroid + RASB, RASB	52	Remission: proteinuria < 1.0 g/dResistance: proteinuria > 1.0 g/d	9
**Katafuchi R 2016** [15]	IgAN with proteinuria 1.0 - 3.5 g/d	59	Tonsillectom + steroid, Steroid	12	Remission: proteinuria < 0.3 g/g or proteinuria < 0.3 g/d	8

N, number; IgAN, immunoglobulin A nephropathy; IS, immunosuppression; RASB, rennin angiotensin system blockade; NS, nephritic syndrome; MMF, mycophenolate mofetil

The quality assessment of the primary studies was presented in Table 1. All studies were considered have a low risk of bias on the issue of incomplete outcome and selective reporting.

### The Oxford classification pathologic lesions response to steroid

#### Mesangial hypercellularity

**Figure 2:**
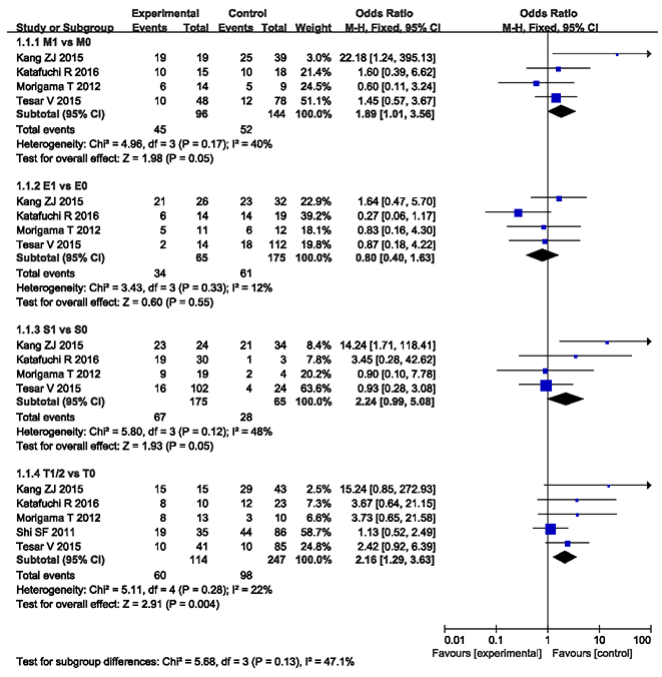
The Oxford classification (MEST) pathologic lesions response to steroid therapy in the patients with IgA nephropathy

In patients with IgAN, M1 (defined as mesangial hypercellularity score > 0.5) had a 1.89-fold [95% confidence interval (CI) 1.01 - 3.56, *P* < 0.05] greater probability of resistance to steroid than M0, which was defined as mesangial hypercellularity score ≤ 0.5 with no evidence of heterogeneity (*I^2^* = 40%, *P* = 0.17) as demonstrated in Figure 2.

#### Endocapillary hypercellularity

95 of the 240 participants were resistant to steroid events reported the prediction of E lesions to steroid therapy. The pooled analysis suggested no difference in steroid sensitivity (OR 0.80, 95% *CI* 0.40 - 1.63, *P* = 0.55) between E1 and E0. No evidence of heterogeneity lay in the included studies (*I^2^* = 12%, *P* = 0.33, Figure 2).

#### Segmental glomerulosclerosis

Resistance to steroid therapy was reported in 95 of the 240 patients with an oxford score S1. No evidence of heterogeneity lay in the included studies (*I^2^* = 48%, *P* = 0.12), as shown in Figure 2. Then the fixed-effect model was used to pool results. The pooled analysis suggested the difference in steroid resistance (OR 2.24, 95% *CI* 0.99 - 5.08, *P* = 0.05) when compared S1, which was defined as the presence of segmental glomerulosclerosis with S0, which was defined as the absence of segmental glomerulosclerosis.

#### Tubular atrophy and interstitial fibrosis

Five studies with 361 participants and 158 end point events reported the association of T with steroid/immunosupression therapy to IgAN. These studies showed that a score of T1/2 (defined as > 25% tubular atrophy/interstitial fibrosis) was associated strongly with the prediction to steroid/immunosupression resistance [T0 (defined as ≤ 25% tubular atrophy/interstitial fibrosis) as reference, OR 2.16, 95% *CI* 1.29 - 3.63, *P* = 0.004], with no evidence of heterogeneity (*I^2^* = 22%, *P* = 0.28, Figure 2).

### MEST lesions compared with each other

**Figure 3:**
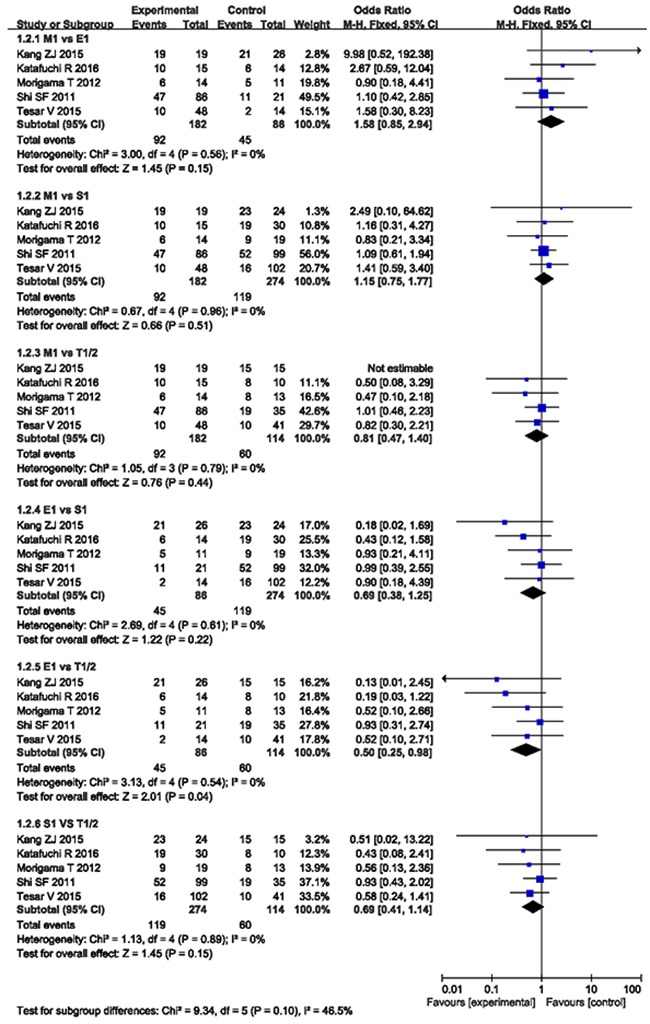
MEST lesions compared with each other from 5 studies

The pooled OR, when compared M1 with E1, S1, and T1/2, were 1.58 (*CI* 0.85 - 2.94, *P* = 0.15), 1.15 (*CI* 0.75 - 1.77, *P* = 0.51), and 0.81 (*CI* 0.47 - 1.40; *P* = 0.44), respectively. The pooled OR of E1 compared with S1, T1/2, and S1 *vs*. T1/2 were 0.69 (*CI* 0.38 - 1.25, *P* = 0.22), 0.50 (*CI* 0.25 - 0.98, *P* = 0.04), and 0.69 (*CI* 0.41 - 1.14, *P* = 0.15), as demonstrated in Figure 3.

### Sensitivity analysis and publication bias

**Figure 4:**
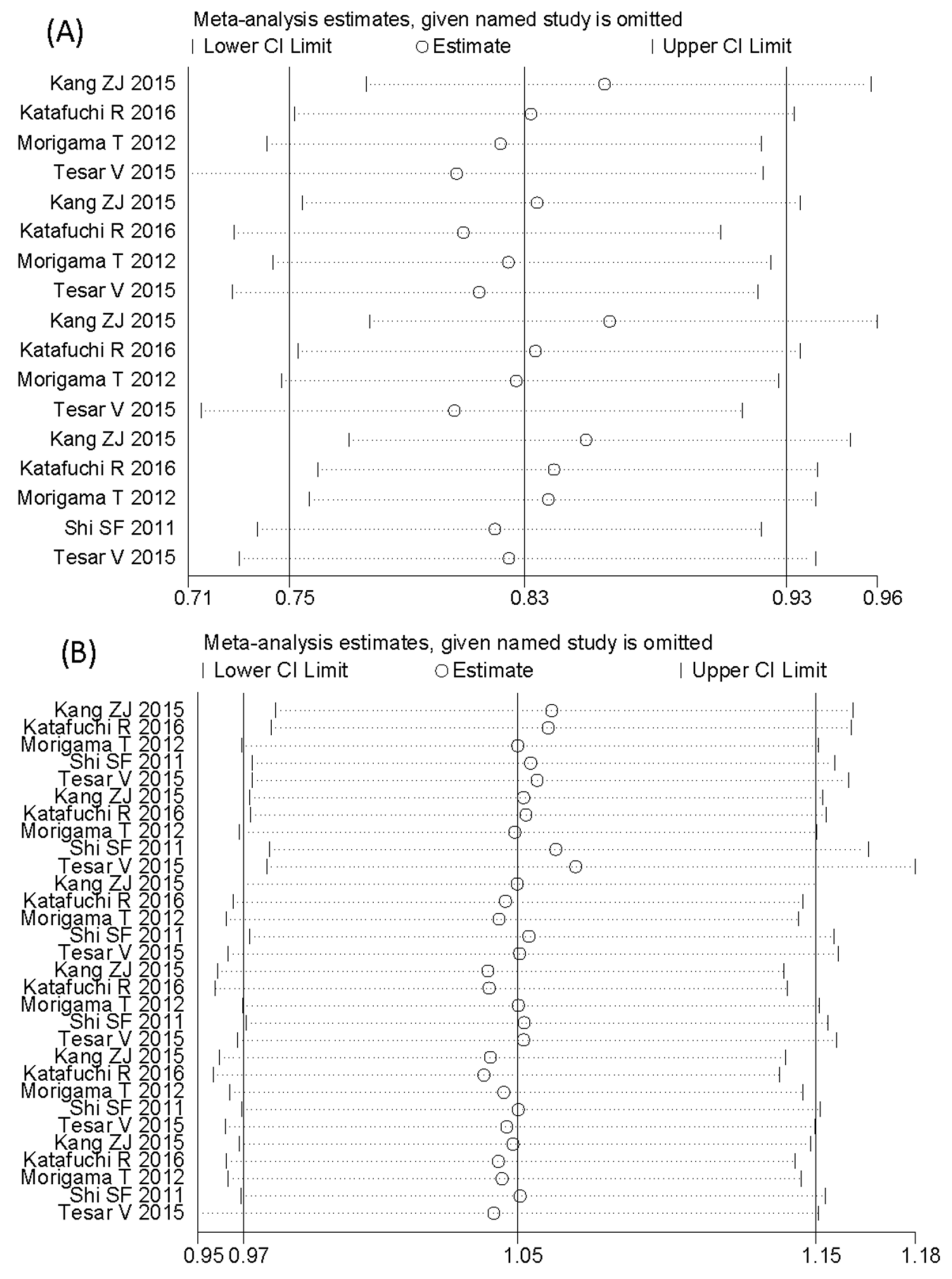
Sensitivity analysis of the meta-analysis **(A)**, Sensitivity analysis shows the meta-analysis is low sensitivity and satisfied stability in the Oxford classification pathologic lesions responded to steroid therapy in patients with IgA nephropathy. **(B)**, Sensitivity analysis shows the meta-analysis is low sensitivity and satisfied stability in MEST lesions compared with each other from 5 studies.

**Figure 5:**
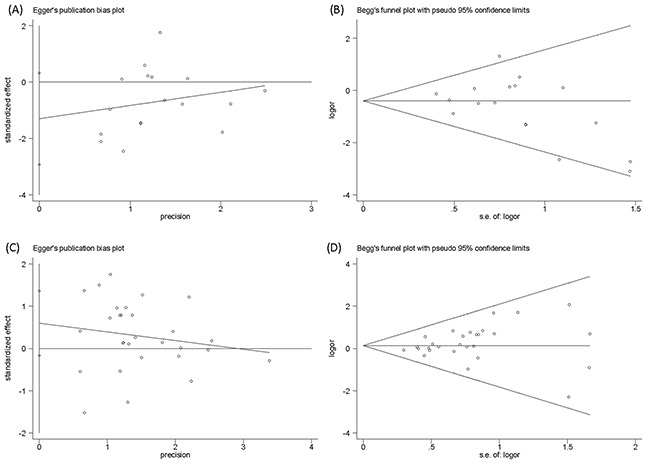
Publication bias of Egger's test and Begg's Test funnel plot **(A, B)**, The Egger's test linear regression test and Begg's rank correlation test provided no evidence of substantial publication bias in the Oxford classification pathologic lesions responded to steroid therapy in patients with IgA nephropathy. **(C, D)**, The Egger's test and Begg's test provided no evidence of substantial publication bias in MEST lesions compared with each other from 5 studies.

Our analysis was stable in the choice of fixed effect models. The fixed effect was used to pool results with no evidence of heterogeneity (*I^2^* < 50%, *P* > 0.10). Sensitivity analysis indicated that the meta-analysis was low sensitivity and high stability (Figure 4). Egger's test and Begg's Test funnel plot were used to explore the publication bias. The Egger's test linear regression test (*P* > 0.066) and Begg's rank correlation test (Pr > |z| = 0.092) provided no evidence of substantial publication bias in the Oxford classification pathologic lesions responded to steroid therapy in patients with IgA nephropathy (Figure 5A, 5B). The Egger's test linear regression test (*P* > 0.144) and Begg's rank correlation test (Pr > |z| = 0.175) provided no evidence of substantial publication bias in MEST lesions compared with each other from 5 studies (Figure 5C, 5D).

## DISCUSSION

Lai et al. [18] reported a RCT with steroid therapy, which demonstrated that IgAN patients with nephrotic syndrome and minimal to mild pathological changes were sensitive to 4-month steroid therapy (40 - 60 mg/d oral prednisolone for first 2 months, and 20 - 30 mg/d for latter 2 months). Other studies have also showed the good response to steroid in patients with minimal histopathological changes and resistant to steroid therapy with severe pathological change [19–21]. Kang Z et al. found that patients with M1, S1, and T1/2 were resistant to steroid therapy [14], however the results have not been validated in the Oxford classification. To our knowledge, it was the first meta-analysis that found the presence of the M1, S1, or T1/2 lesions in the Oxford Classification was strongly associated with the resistance to steroid/immunosuppressive therapy.

Lesion E of indicators for predictive value and immunosuppressive therapy was the most controversial. A meta-analysis suggested that lesion E was not associated with the progression to the worsening of renal function [9], while the Oxford Classification work group indicated that lesion E was mainly responsible for the response to immunosuppressive therapy, most frequently corticosteroids, but not for the prediction on renal function [8, 9]. However, in a cohort study receiving no immunosuppression therapy, E was found to be the independent predictive factor of progression to renal failure [22], but not related to the response to steroid therapy (*P* = 0.318) [14]. These studies suggested that the use of immunosuppression may mask the predictive value of lesion E in renal outcomes. Although these findings do not in themselves support the routine use of immunosuppression when the E lesions are present, they do justify a prospective trial of immunosuppression in IgAN with the E lesions [23]. In the present meta-analysis, although E1 was not associated with the response to steroid therapy when compared with E0, we found that E1 was related to the higher probability of proteinuria remission and a lower resistance to steroid/immunosuppressive therapy than T1/2 (OR 0.50, *CI* 0.25 - 0.98, *P* = 0.04).

A recent publication reviewed S lesions in the Oxford Classification subject cohort and correlated histology with clinical presentation and outcome. In a retrospective series of 1147 subjects from 13 European countries, the VALIGA (European Validation Study of the Oxford Classification of IgAN) study demonstrated the S score to be an independent predictor of outcome in IgAN [24]. A review of S lesions indicates there may be clinical utility in the subclassification of segmental sclerosis, which identified those cases with evidence of podocyte damage [23]. Katafuchi R et al. suggested that only pathologic lesion S showed a significant heterogeneity between S0 and S1 to steroid treatment in the disappearance of proteinuria (*P* = 0.045) [15]. Our meta-analysis suggested the difference in steroid resistance (OR 2.24, 95% *CI* 0.99 - 5.08, *P* = 0.05) when compared S1 with S0.

Pathologic lesion T was a consistent, independent predictor of renal outcomes, with more variable results for M and S lesions [23]. The T score mostly reflects the stage of the disease at the time of biopsy, and those participants with more advanced chronic damage have a shorter time to ESRD [23, 25]. This meta-analysis indicates that a score of T1/2 was associated strongly with the prediction to steroid/immunosupression resistance.

There is much controversy about the role of crescents as a significant prognostic factor in IgAN. In a larger broadly based cohort than in the original Oxford research, crescents are predictive of outcome, and they recommended that crescents be added to the MEST score, and biopsy reporting should provide a MEST-C score [23]. A multicenter cohort study pooled from four retrospective studies indicated the following crescent scores to the Oxford Classification: C0 (no crescent) and C1 (≤ 25% crescents) identified patients at increased risk of poor outcome without immunosuppression, and C2 (> 25% crescents) identified patients at even greater risk of progression, even with immunosuppression [26]. The efficacy of steroid and cyclophosphamide pulses therapy in crescentic glomerulonephritis might be affected by reduction of glomerular chemokine expression and stabilized renal function [27–29]. However, limited evidences from IgAN patients with crescents should be further investigated to update the response of Oxford classification to steroid treatment.

The main limitation of this meta-analysis is that the definition of remission and no response in the included studies was not in consistence. The included studies and participants were too small. Since there were only 5 studies with 637 patients were included in this study. Therefore, high-quality clinical trials with a large sample size are needed to define the response of Oxford classification to steroid therapy in patients with IgAN.

In conclusions, IgA nephropathy patients with serious pathological changes (M1, S1, and T1/2) were more resistant to steroid than slight ones (M0, S0, and T0). Patients with M1, S1, and T1/2 were resistant to steroid therapy, and E1 is better response to steroid therapy than T1/2.

## MATERIALS AND METHODS

### Data sources and search strategy

We performed a systematic review of the published researches according to the approach recommended by the PRISMA (Preferred Reporting Items for Systematic Reviews and Meta-Analyses) guidelines for the conduct of meta-analyses without any language restriction [12]. We searched Chinese Biomedical Database, EMBASE, Cochrane Library, MEDLINE and PubMed for articles from 2009 to December 2016 with key words and Medical Subject Headings that covered “IgAN” or “IgA nephropathy” or “immunoglobulin A nephropathy” or “IgA nephritis” and “Oxford Classification” or “Oxford”, and “steroid” or “glucocorticoid” or “corticosteroid” or “prednisolone”. The ClinicalTrials.gov website was also searched for clinical trials that were registered as completed but not yet published. Reference lists from identified trials and review articles were manually scanned to identify related research references at the same time as described in Figure 1.

### Selection criteria

We collected all eligible articles about the relationship between the pathological findings and therapeutic effects in patients with IgAN in this meta-analysis. Inclusion criteria of studies: (a) the original research related to steroid therapy for IgAN, (b) articles provided exact data of clinical remission, and (c) articles with clear comparison of each MEST lesions. Exclusion criteria: (a) studies such as systemic review, case report, comments, conference abstracts, editorials etc, and (b) articles that had no definitions on clinical remission and no response to steroid therapy.

### Data extraction and quality assessment

**Figure 6:**
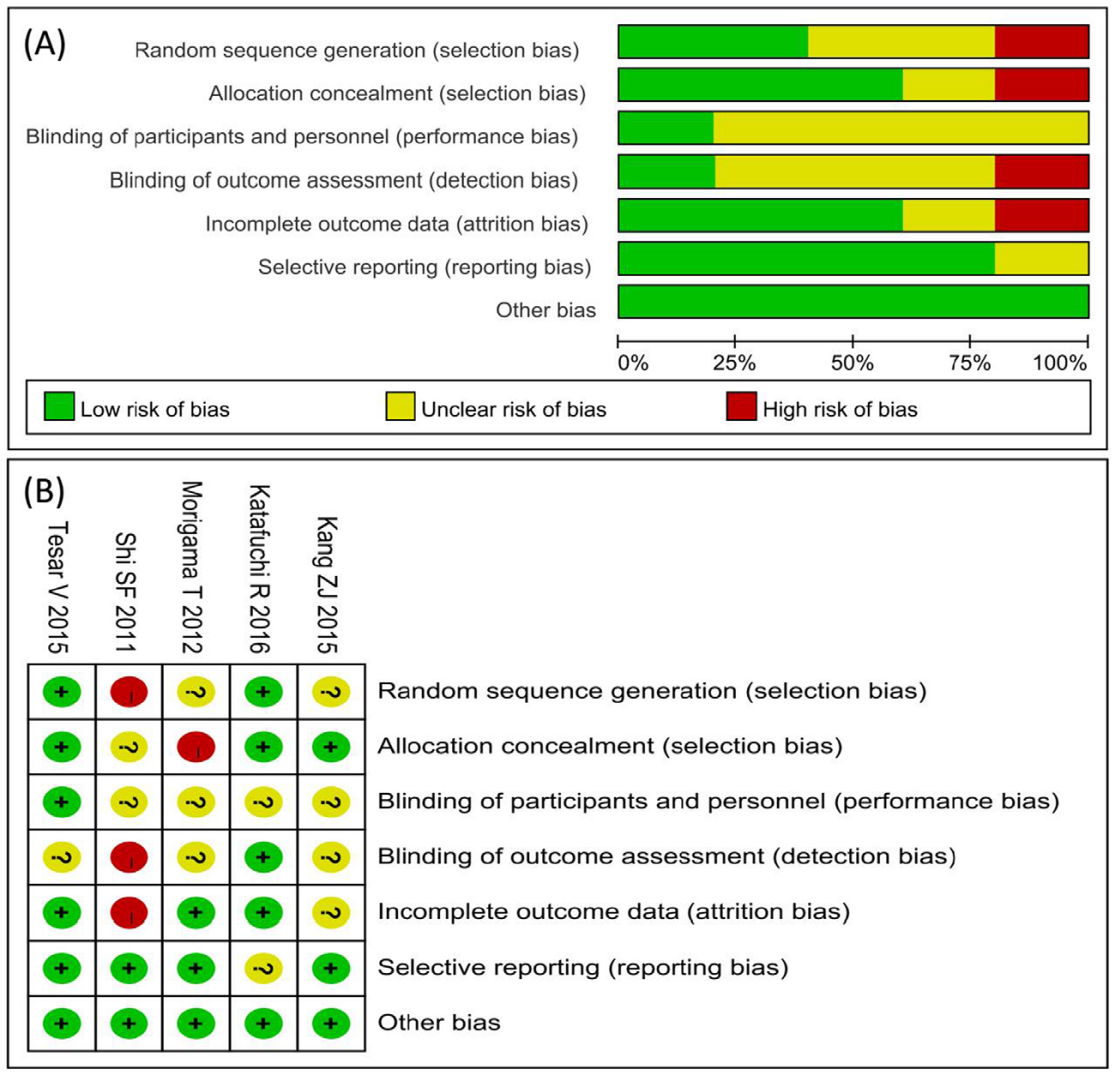
Risk of bias graph **(A)** and risk of bias summary **(B)**.

The article searching, data extraction, and quality assessment were undertaken independently by two investigators (Pingping Yang and Xi Chen) with a standardized approach, and disagreements were resolved through discussions or referral to a third author (Gaosi Xu). All potentially eligible citations that we had searched were examined in detail to identify studies that satisfied the criterion. The extracted data included the first author's name, year of publication, follow-up duration, definition about disappearance of proteinuria or clinical remission, steroid doses and modalities of treatment, number of patients receiving steroid, response to steroid therapy, and the benefits of therapy in the presence or absence of the Oxford Classification pathologic lesions (M, E, S, and T, Table 1). The RCTs and cohort studies quality assessment was completed by using Review Manager 5.3 (Cochrane collaboration, Oxford, UK) risk of bias tool including four sections: selection, performance, detection, attrition, reporting, and other bias as shown in Figure 6. The Newcastle-Ottawa Scale ranged 0 to 9 score was used to evaluate the cohort study quality displayed in Table 1.

### Data synthesis and analysis

The data were abstracted and analyzed with Review Manager 5.3 and Stata 12.0 (Stata Corporation, TX, USA) to make the outcomes more convinced. Results are expressed as odds ratio (OR) with 95% confidence intervals (CI) obtained by a fix effects model using the DerSimonian and Laird method. Value of OR < 1 indicates a reduction in risk for outcome with the experimental treatment. On the contrary, value of OR > 1 indicates an increase in risk. OR random-effects model was used to deal with data in light of the heterogeneity in results and study clinical characteristics while the fix effects model was poor of heterogeneity. We used the *I^2^* test to estimate the heterogeneity across trials, with *P* < 0.1 being considered significant. We considered a *P* value of not more than 0.05 to be significant. Subgroup and sensitivity analyses were used to explore the potential sources of heterogeneity. Potential publication biases were assessed graphically by using the Egger's test and Begg's Test funnel plot.

## Abbreviations

IgAN: Immunoglobulin A nephropathy
ESRD: end-stage renal failure
M: mesangial hypercellularity
E: endocapillary hypercellularity
S: segmental glomerulosclerosis
T: tubular atrophy and interstitial fibrosis
KDIGO: The Kidney Disease Improving Global Outcomes
eGFR: estimated glomerular filtration rate
RCTs: randomized controlled trials
PRISMA: Preferred Reporting Items for Systematic Reviews and Meta-Analyses
OR: odds ratio
CI: confidence intervals
